# Differentiable PAC–Bayes Objectives with Partially Aggregated Neural Networks

**DOI:** 10.3390/e23101280

**Published:** 2021-09-29

**Authors:** Felix Biggs, Benjamin Guedj

**Affiliations:** 1 Centre for Artificial Intelligence, Department of Computer Science, University College London, London WC1V 6LJ, UK; fbiggs@cs.ucl.ac.uk; 2 Inria Lille—Nord Europe Research Centre and Inria London, 59800 Lille, France

**Keywords:** statistical learning theory, PAC–Bayes theory, deep learning

## Abstract

We make two related contributions motivated by the challenge of training stochastic neural networks, particularly in a PAC–Bayesian setting: (1) we show how averaging over an ensemble of stochastic neural networks enables a new class of partially-aggregated estimators, proving that these lead to unbiased lower-variance output and gradient estimators; (2) we reformulate a PAC–Bayesian bound for signed-*output* networks to derive in combination with the above a directly optimisable, differentiable objective and a generalisation guarantee, without using a surrogate loss or loosening the bound. We show empirically that this leads to competitive generalisation guarantees and compares favourably to other methods for training such networks. Finally, we note that the above leads to a simpler PAC–Bayesian training scheme for sign-*activation* networks than previous work.

## 1. Introduction

The use of stochastic neural networks has become widespread in the PAC–Bayesian and Bayesian deep learning [1] literature as a way to quantify predictive uncertainty and obtain generalisation bounds. PAC–Bayesian theorems generally bound the expected loss of *randomised* estimators, so it has proven easier to obtain non-vacuous numerical guarantees on generalisation in such networks.

However, we observe that when training these in the PAC–Bayesian setting, the objective used is generally somewhat divorced from the bound on misclassification loss itself, often because non-differentiability leads to difficulties with direct optimisation. For example, Langford and Caruana [2], Zhou et al. [3], and Dziugaite and Roy [4] all initially train non-stochastic networks before using them as the mode of a distribution, with variance chosen, respectively, through a computationally-expensive sensitivity analysis, as a proportion of weight norms, or by optimising an objective with both a surrogate loss function and a different dependence on the Kullback–Leibler (KL) divergence from their bound.

In exploring methods to circumvent this gap, we also note that PAC–Bayesian bounds can often be straightforwardly adapted to aggregates or averages of estimators, leading directly to analytic and differentiable objective functions (for example, [5]). Unfortunately, averages over deep stochastic networks are usually intractable or, if possible, very costly (as found by [6]).

Motivated by these observations, our main contribution is to obtain a compromise by defining new and general “*partially-aggregated*” Monte Carlo estimators for the average output and gradients of deep stochastic networks (Section 3), with the direct optimisation of PAC–Bayesian bounds in mind. Although our main focus here is on the use of this estimator in a PAC–Bayesian application, we emphasise that the technique applies generally to stochastic networks and thus has links to other variance-reduction techniques for training them, such as the pathwise estimator used in the context of neural networks by [7] amongst many others or Flipout [8]; indeed, it can be used in combination with these techniques. We provide proofs (Section 4) that this application leads to lower variances than a Monte Carlo forward pass and lower variance final-layer gradients than REINFORCE [9].

A further contribution of ours is a first application of this general estimator to non-differentiable “signed-output” networks (with a final output ∈{−1,+1} and arbitrarily complex other structure, see Section 4). As well as reducing variances as stated above, a small amount of additional structure in combination with partial-aggregation enables us to extend the pathwise estimator to the other layers, which usually requires a fully differentiable network and eases training by reducing the variance of gradient estimates.

We adapt a binary classification bound (Section 5) from Catoni [10] to these networks, yielding straightforward and directly differentiable objectives when used in combination with aggregation. Closing this gap between objectives and bounds leads to improved theoretical properties.

Further, since most of the existing PAC–Bayes bounds for neural networks have a heavy dependency on the distance from initialisation of the obtained solution, we would intuitively expect these lower variances to lead to faster convergence and tighter bounds (from finding low-error solutions nearer to the initialisation). We indeed observe this experimentally, showing that training PAC–Bayesian objectives in combination with partial aggregation leads to competitive experimental generalisation guarantees (Section 6), and improves upon naive Monte Carlo and REINFORCE.

As a useful corollary, this application also leads us to a similar but simpler PAC–Bayesian training method for sign-activation neural networks than Letarte et al. [6], which successfully aggregated networks with all sign activation functions ∈{+1,−1} and a non-standard tree structure, but incurred an exponential KL divergence penalty and a heavy computational cost (so that in practice they often resorted to a Monte Carlo estimate). Further, the lower variance of our obtained estimator predictions enables us to use the Gibbs estimator directly (where we draw a single sample function for every new example), leading to a modified bound on the misclassification loss which is a factor of two tighter without a significant performance penalty.

We discuss further and outline future work in Section 7.

## 2. Background

We begin here by setting out our notation and the requisite background.

Generally, we consider parameterised functions, $\left\{f_{\theta }:X\to Y|\theta \in \Theta \subset R^{N}\right\}$, in a specific form, choosing $X\subset R^{d_{0}}$ and an arbitrary output space Y which could be for example {−1,+1} or R. We wish to find functions minimizing the out-of-sample risk, $R\left(f\right)=E_{(x,y)∼D}\ell \left(f\left(x\right),y\right)$, for some loss function *ℓ*, for example the 0-1 misclassification loss for classification, $\ell_{0-1}\left(y,y^{\prime }\right)=1\left\{y\neq y^{\prime }\right\}$, or the binary linear loss, $\ell_{\mathrm{lin}}\left(y,y^{\prime }\right)=\frac{1}{2}\left(1-yy^{\prime }\right)$, with Y={+1,−1}. These must be chosen based on an i.i.d. sample $S={\left\{\left(x_{i},y_{i}\right)\right\}}_{i=1}^{m}∼D^{m}$ from the data distribution D, using the surrogate of in-sample empirical risk, $R_{S}\left(f\right)=\frac{1}{m}\sum_{i=1}^{m}\ell \left(f\left(x_{i}\right),y_{i}\right)$. We denote the expected and empirical risks under the misclassification and linear losses, respectively $R^{0-1},R^{\mathrm{lin}},R_{S}^{0-1}$ and $R_{S}^{\mathrm{lin}}$.

In this paper, we consider learning a distribution (PAC–Bayesian posterior), *Q*, over the parameters θ. PAC–Bayesian theorems then provide bounds on the expected generalization risk of randomised classifiers, where every prediction is made using a newly sampled function from our posterior, $f_{\theta },\;\theta ∼Q$.

We also consider averaging the above to obtain *Q*-aggregated prediction functions,

(1) $$F_{Q}\left(x\right):=E_{\theta ∼Q}f_{\theta }\left(x\right).$$

In the case of a convex loss function, Jensen’s inequality lower bounds the risk of the randomised function by its *Q*-aggregate: $\ell \left(F_{Q}\left(x\right),y\right)\leq E_{f∼Q}\ell \left(f\left(x\right),y\right)$. The equality is achieved by the linear loss, a fact we will exploit to obtain an easier PAC–Bayesian optimisation objective in Section 5.

### 2.1. Analytic Q-Aggregates for Signed Linear Functions

*Q*-aggregate predictors are analytically tractable for “signed-output” functions (here the sign function and “signed” functions have outputs ∈{+1,−1}, as the terminology “binary”, used sometimes in the literature, suggests to us too strongly an output ∈{0,1}) of the form $f_{w}\left(x\right)=\mathrm{sign}\left(w\cdot x\right)$ under a normal distribution, Q(w)=N(μ,I), as specifically considered in a PAC–Bayesian context for binary classification by [5], obtaining an differentiable objective similar to the SVM. Provided x≠0:

(2) $$F_{Q}\left(x\right):=E_{w∼N(\mu ,I)}\mathrm{sign}\left(w\cdot x\right)=\mathrm{erf}\left(\frac{\mu \cdot x}{\sqrt{2}∥x∥}\right).$$

In Section 4, we will consider aggregating signed output (f(x)∈{+1,−1}) functions of a more general form.

### 2.2. Monte Carlo Estimators for More Complex Q-Aggregates

The framework of *Q*-aggregates can be extended to less tractable cases (for example, with $f_{\theta }$ a randomised or a “Bayesian” neural network, see, e.g., [1]) through a simple and unbiased Monte Carlo approximation:

(3) $$F_{Q}\left(x\right)=E_{\theta ∼Q}f_{\theta }\left(x\right)\approx \frac{1}{T}\sum_{t=1}^{T}f_{\theta^{t}}\left(x\right):={\hat{F}}_{Q}\left(x\right).$$

If we go on to parameterize our posterior *Q* by $\phi \in \Phi \subset R^{N}$ as $Q_{\phi }$ and wish to obtain gradients without a closed form for $F_{Q_{\phi }}\left(x\right)=E_{\theta ∼Q_{\phi }}f_{\theta }\left(x\right)$, there are two possibilities. One is REINFORCE [9], which requires only a differentiable density function, $q_{\phi }\left(\theta \right)$ and makes a Monte Carlo approximation to the left hand side of the identity $\nabla_{\phi }E_{\theta ∼q_{\phi }}f_{\theta }\left(x\right)=E_{\theta ∼q_{\phi }}\left[f_{\theta }\left(x\right)\nabla_{\phi }\log q_{\phi }\left(\theta \right)\right]$.

The other is the pathwise estimator, which additionally requires that $f_{\theta }\left(x\right)$ be differentiable w.r.t. θ, and that the probability distribution chosen has a standardization function, $S_{\phi }$, which removes the ϕ dependence, turning a parameterised $q_{\phi }$ into a non-parameterised distribution *p*: for example, $S_{\mu ,\sigma }\left(X\right)=\left(X-\mu \right)/\sigma$ to transform a general normal distribution into a standard normal. If this exists, the right hand side of $\nabla_{\phi }E_{\theta ∼q_{\phi }}f_{\theta }\left(x\right)=E_{\epsilon ∼p}\nabla_{\phi }f_{S_{\phi }^{-1}\left(\epsilon \right)}\left(x\right)$ generally yields lower-variance estimates than REINFORCE (see for a modern survey [11]).

The variance introduced by REINFORCE can make it difficult to train neural networks when the pathwise estimator is not available, for example when non-differentiable activation functions are used. Below we find a compromise between the analytically closed form of (2) and the above estimator that enables us to make differentiable certain classes of network and extend the pathwise estimator where otherwise it could not be used. Through this we are able to stably train a new class of network.

### 2.3. PAC–Bayesian Approach

We use PAC–Bayes in this paper to obtain generalisation guarantees and theoretically-motivated training methods. The primary bound utilised is based on the following theorem, valid for a loss taking values in [0,1]:

**Theorem** **1** ([10], Theorem 1.2.6). *Given probability measure P on hypothesis space F and α>1, for all Q on F with probability at least 1−δ over $S∼D^{m}$,*

$$E_{f∼Q}R\left(f\right)\leq \underset{\lambda >1}{\inf}\Phi_{\lambda /m}^{-1}\left[E_{f∼Q}R_{S}\left(f\right)+\frac{\alpha }{\lambda }\Delta \right]$$

*with $\Phi_{\gamma }^{-1}\left(t\right)=\frac{1-\exp(-\gamma t)}{1-\exp(-\gamma )}$ and $\Delta =\mathrm{KL}\left(Q|P\right)-\log\delta +2\log\left(\frac{\log\alpha^{2}\lambda }{\log\alpha }\right)$.*

This slightly opaque formulation (used previously by [3]) gives essentially identical results when KL/m is large to the better-known “small-kl” PAC–Bayes bounds originated by Langford and Seeger [12], Seeger et al. [13]. It is chosen because it leads to objectives that are *linear* in the empirical loss and KL divergence, like

(4) $$E_{f∼Q}R_{S}\left(f\right)+\frac{\mathrm{KL}\left(Q|P\right)}{\lambda }.$$

This objective is minimised by a Gibbs distribution and is closely related to the evidence lower bound (ELBO) usually optimised by Bayesian Neural Networks [1]. Such a connection has been noted throughout the PAC–Bayesian literature; we refer the reader to [14] or [15] for a formalised treatment.

## 3. The Partial Aggregation Estimator

Here we outline our main contribution: a reformulation of *Q*-aggregation for neural networks leading to different, lower-variance, Monte Carlo estimators for their outputs and gradients. These estimators apply to networks with a dense final layer, and arbitrary stochastic other structure (for example convolutions, residual layers or a non-feedforward structure). Specifically, they take the form

(5) $$f_{\theta }\left(x\right)=A\left(w\cdot \eta_{\theta^{\neg w}}\left(x\right)\right)$$

with $\theta =\mathrm{vec}\left(w,\theta^{\neg w}\right)\in \Theta \subset R^{D}$, $w\in R^{d}$, and $\theta^{\neg w}\in \Theta^{\neg w}\subset R^{D-d}$ the parameter set excluding w, for the non-final layers of the network. These non-final layers are included in $\eta_{\theta^{\neg w}}:X\to A^{d}\subseteq R^{d}$ and the final activation is A:R→Y. For simplicity we have used a one-dimensional output but we note that the formulation and below derivations trivially extend to a vector-valued function with elementwise activations. We require the distribution over parameters to factorise like $Q\left(\theta \right)=Q^{w}\left(w\right)Q^{\neg w}\left(\theta^{\neg w}\right)$, which is consistent with the literature.

We recover a similar functional form to that considered in Section 2.1 by rewriting the function as A(w·a) with $a\in A^{d}$ the randomised hidden-layer activations. The “aggregated” activation function on the final layer, which we define as $I\left(a\right):=\int A\left(w\cdot a\right)\;dQ^{w}\left(w\right)$, may then be analytically tractable. For example, with w∼N(μ,I) and a sign final activation, we recall (2) where $I\left(a\right)=\mathrm{erf}\left(\frac{\mu \cdot a}{\sqrt{2}∥a∥}\right)$.

Using these definitions we can write the *Q*-aggregate in terms of the conditional distribution on the activations, a, which takes the form ${\tilde{Q}}^{\neg w}\left(a|x\right):=\left(\eta_{\left(\cdot \right)}\left(x\right)\right)\circ Q^{\neg w}$, (i.e., the distribution of $\eta_{\theta^{\neg w}}\left(x\right)|x$, with $\theta^{\neg w}∼Q^{\neg w}$). The *Q*-aggregate can then be stated as

$$\begin{array}{cc} F_{Q}\left(x\right) & :=E_{\theta ∼Q}\left[f_{\theta }\left(x\right)\right]\\ \; & =\int_{\theta^{\neg w}}\left[\int_{R^{d}}A\left(w\cdot \eta_{\theta^{\neg w}}\left(x\right)\right)\;dQ^{w}\left(w\right)\right]\;dQ^{\neg w}\left(\theta^{\neg w}\right)\\ \; & =\int_{\theta^{\neg w}}I\left(\eta_{\theta^{\neg w}}\left(x\right)\right)\;dQ^{\neg w}\left(\theta^{\neg w}\right)\\ \; & =\int_{A^{d}}I\left(a\right)\;d\left\{\left(\eta_{\left(\cdot \right)}\left(x\right)\right)\circ Q^{\neg w}\right\}\left(a\right)\\ \; & =:\int_{A^{d}}I\left(a\right)\;d{\tilde{Q}}^{\neg w}\left(a|x\right). \end{array}$$

In most cases, the final integral cannot be calculated exactly or involves a large summation, so we resort to a Monte Carlo estimate, for each x drawing *T* samples of the randomised activations, ${\left\{a^{t}\right\}}_{t=1}^{T}∼{\tilde{Q}}^{\neg w}\left(a|x\right)$ to obtain the “partially-aggregated” estimator

(6) $$F_{Q}\left(x\right)=\int_{A^{d}}I\left(a\right)\;d{\tilde{Q}}^{\neg w}\left(a|x\right)\approx \frac{1}{T}\sum_{t=1}^{T}I\left(a^{t}\right)={\hat{F}}_{Q}^{*}\left(x\right).$$

This is quite similar to the original estimator from (3), but in fact the aggregation of the final layer may significantly reduce the variance of the outputs and also make better gradient estimates possible, as we will show below.

### 3.1. Reduced Variance Estimates

**Proposition** **1.** Lower variance outputs: *For a neural network as defined by Equation (5) and the unbiased Q-aggregation estimators defined by Equations (3) and (6),*

$$V_{Q}\left[{\hat{F}}_{Q}^{*}\left(x\right)\right]\leq V_{Q}\left[{\hat{F}}_{Q}\left(x\right)\right].$$

**Proof.**  Treating a as a random variable, always conditioned on x, we have

$$\begin{array}{cc} \; & V_{Q}\left[{\hat{F}}_{Q}\left(x\right)\right]-V_{Q}\left[{\hat{F}}_{Q}^{☆}\left(x\right)\right]=E_{Q}|{\hat{F}}_{Q}{\left(x\right)|}^{2}-E_{Q}{\left|{\hat{F}}_{Q}^{☆}\left(x\right)\right|}^{2}\\ \; & =\frac{1}{T}E_{a|x}\left[E_{w}{\left|A\left(w\cdot a\right)\right|}^{2}-{\left|E_{w}A\left(w\cdot a\right)\right|}^{2}\right]\\ \; & =\frac{1}{T}E_{a|x}\left[V_{w}\left[A\left(w\cdot a\right)\right]\right]\geq 0. \end{array}$$

 □

From the above we see that the aggregate outputs estimated through partial-aggregation have lower variances. Next, we consider the two unbiased gradient estimators for the distribution over final-layer weights, w, arising from partial-aggregation or REINFORCE (as would be used, for example, where the final layer is non-differentiable). Assuming $Q^{w}$ has a density, $q_{\phi }\left(\theta^{w}\right)$, parameterised by ϕ, these use forward samples of ${\left\{w^{t},\theta^{\neg w,(t)}\right\}}_{t=1}^{T}$ as:

$$\begin{array}{cc} \hat{G}\left(x\right) & :=\frac{1}{T}\sum_{t=1}^{T}A\left(w^{t}\cdot \eta^{t}\right)\nabla_{\phi }\log q_{\phi }\left(w^{t}\right)\\ {\hat{G}}^{*}\left(x\right) & :=\frac{1}{T}\sum_{t=1}^{T}\nabla_{\phi }I_{q_{\phi }}\left(\eta^{t}\right). \end{array}$$

**Proposition** **2.** Lower variance gradients: *Under the conditions of Proposition 1 and the above definitions,*

$${\mathrm{Cov}}_{Q}\left[{\hat{G}}^{*}\left(x\right)\right]⪯{\mathrm{Cov}}_{Q}\left[\hat{G}\left(x\right)\right]$$

*where A⪯B⇔B−A is positive semi-definite. Thus, for all u≠0, $V\left[{\hat{G}}^{*}\left(x\right)\cdot u\right]\leq V\left[\hat{G}\left(x\right)\cdot u\right]$.*

**Proof.**  Writing $v:=\nabla_{\phi }\log q_{\phi }\left(w\right)$ and using the unbiasedness of the estimators,

$$\begin{array}{cc} \; & {\mathrm{Cov}}_{Q}\left[{\hat{G}}_{Q}\left(x\right)\right]-{\mathrm{Cov}}_{Q}\left[{\hat{G}}_{Q}^{☆}\left(x\right)\right]\\ \; & =E_{Q}\left[{\hat{G}}_{Q}\left(x\right){\hat{G}}_{Q}{\left(x\right)}^{T}\right]-E_{Q}\left[{\hat{G}}_{Q}^{☆}\left(x\right){\hat{G}}_{Q}^{☆}{\left(x\right)}^{T}\right]\\ \; & =\frac{1}{T}E_{a|x}\left[E_{w}\left[A{\left(w\cdot a\right)}^{2}vv^{T}\right]-\nabla_{\phi }I_{q_{\phi }}\left(\eta^{t}\right)\left(\nabla_{\phi }I_{q_{\phi }}{\left(\eta^{t}\right)}^{T}\right)\right]\\ \; & =\frac{1}{T}E_{a|x}\left[{\mathrm{Cov}}_{w}\left[A\left(w\cdot a\right)\nabla_{\phi }\log q_{\phi }\left(w\right)\right]\right]⪰0 \end{array}$$

where in the final line we have used that $\nabla_{\phi }I_{q_{\phi }}\left(\eta^{t}\right)=\nabla_{\phi }E_{w}\left[A\left(w\cdot \eta^{t}\right)\right]=E_{w}\left[A\left(w\cdot \eta^{t}\right)v\right]$. □

### 3.2. Single Hidden Layer

For clarity (and to introduce notation to be used in Section 4.2) we will briefly consider the case of a neural network with one hidden layer, $f_{\theta }\left(x\right)=A_{2}\left(w_{2}\cdot A_{1}\left(W_{1}x\right)\right)$. The randomised parameters are $\theta =\mathrm{vec}(w_{2},W_{1})$, $W_{1}\in R^{d_{1}\times d_{0}}$, $w_{2}\in R^{d_{1}}$ and the elementwise activations are $A_{1}:R^{d_{1}}\to A_{1}^{d_{1}}\subseteq R^{d_{1}}$ and $A_{2}:R\to Y$. We choose the distribution $Q\left(\theta \right)=:Q_{2}\left(w_{2}\right)Q_{1}\left(W_{1}\right)$ to factorise over the layers. This is identical to the above and sets $\eta_{W_{1}}\left(x\right)=A_{1}\left(W_{1}x\right)$.

Sampling a is straightforward if sampling $W_{1}$ is. Further, if the final layer aggregate is differentiable, and so is the hidden layer activation $A_{1}$, we may be able to use the lower-variance pathwise gradient estimator for gradients with respect to $Q_{1}$. We note that this may be possible even if the activation $A_{2}$ is not differentiable, as in Section 4, where we extend the pathwise estimator where we could not otherwise use it.

Computationally, we may implement the above by analytically finding the distribution on the “pre-activations” $W_{1}x$ (trivial for the normal distribution) before sampling this and passing through the activation. With the pathwise estimator this is known as the “local reparameterization trick” [16], which can lead to considerable computational savings on parallel minibatches compared to direct hierarchical sampling, $a=A_{1}\left(W_{1}x\right)$ with $W_{1}∼Q_{1}$. We will utilise this in all our reparameterizable dense networks, and a variation on it to save computation when using REINFORCE in Section 4.2 and Section 6.

## 4. Aggregating Signed-Output Networks

Here we consider a first practical application of the aggregation estimator to stochastic neural networks with a final dense sign-activated layer. We have seen above that this partial aggregation leads to better-behaved training objectives and lower-variance gradient estimates across arbitrary other network structure, It may also allow use of pathwise gradients for the other layers, which would not be possible otherwise due to the non-differentiability of the final layer.

Specifically, these networks take the form of Equation (5) with the final layer activation a sign function and weights drawn from a unit variance normal distribution, $Q^{w}\left(w\right)=N\left(\mu ,I\right)$. The aggregate I(a) is given by Equation (2). Normally-distributed weights are chosen because of the simple analytic forms for the aggregate (reminiscent of the tanh activation occasionally used for neural networks) and KL divergence (effectively an $L^{2}$ regularisation penalty); we note however that closed forms are available for other commonly-used distributions such as the Laplace.

Using Equations (3) and (6) with independent samples ${\left\{\left(w^{t},\theta^{\neg w,(t)}\right)\right\}}_{t=1}^{T}∼Q$ and $\eta^{t}:=\eta_{\theta^{\neg w,(t)}}\left(x\right)$ leads to the two unbiased estimators for the output (henceforth assuming the technical condition $P_{\eta |x}\left\{\eta =0\right\}=0$ that allows aggregation to be well-defined).

(7) $$\begin{array}{cc} {\hat{F}}_{Q}\left(x\right) & :=\frac{1}{T}\sum_{t=1}^{T}\mathrm{sign}\left(w^{t}\cdot \eta^{t}\right) \end{array}$$

(8) $$\begin{array}{cc} {\hat{F}}_{Q}^{*}\left(x\right) & :=\frac{1}{T}\sum_{t=1}^{T}\mathrm{erf}\left(\frac{\mu \cdot \eta^{t}}{\sqrt{2}∥\eta^{t}∥}\right). \end{array}$$

It follows immediately from Propositions 1 and 2 that the latter and the associated gradient estimators have lower variances than the former or the REINFORCE gradient estimates (which we would otherwise have to use due to the non-differentiability of the final layer).

### 4.1. Lower Variance Estimates of Aggregated Sign-Output Networks

We clarify the situation with the lower variance estimates further below. In particular, we find that the reduction in variance from using the partial-aggregation estimator is controlled by the norm ∥μ∥, so that for small ∥μ∥ (as could be expected early in training) the difference can be large, while as ∥μ∥ grows, the difference in variance is controlled and we could reasonably revert to the Monte Carlo (or Gibbs) estimator. Note also that as $F_{Q}\left(x\right)\to \pm 1$ (as expected after training), both variances disappear.

We also show that a stricter condition than Proposition 2 holds on the variances of the aggregated gradients here, and thus that the non-aggregated gradients are noisier in all cases than the aggregate.

**Proposition** **3.** *With the definitions given by Equation (7), for all $x\in R^{d_{0}}$, T∈N, and Q with normally-distributed final layer,*

$$0\leq V_{Q}\left[{\hat{F}}_{Q}\left(x\right)\right]-V_{Q}\left[{\hat{F}}_{Q}^{*}\left(x\right)\right]\leq \frac{1}{T}\left(1-{\left|\mathrm{erf}\left(\frac{∥\mu ∥}{\sqrt{2}}\right)\right|}^{2}\right).$$

**Proof.**  The left identity follows directly from Proposition 1. We also have

$$\begin{array}{cc} V_{Q}\left[{\hat{F}}_{Q}\left(x\right)\right]-V_{Q}\left[{\hat{F}}_{Q}^{☆}\left(x\right)\right]\; & =\frac{1}{T}E_{a|x}\left[V_{w}\left[\mathrm{sign}\left(w\cdot a\right)\right]\right]\\ \; & =\frac{1}{T}\left(1-E_{a|x}{\left|\mathrm{erf}\left(\frac{\mu \cdot \eta }{\sqrt{2}∥\eta ∥}\right)\right|}^{2}\right)\\ \; & \leq \frac{1}{T}\left(1-{\left|\mathrm{erf}\left(\frac{∥\mu ∥}{\sqrt{2}}\right)\right|}^{2}\right). \end{array}$$

 □

**Proposition** **4.** *Under the conditions of Proposition 3,*

$$\mathrm{Cov}\left[{\hat{G}}^{*}\left(x\right)\right]⪯\mathrm{Cov}\left[\hat{G}\left(x\right)\right]+\frac{1-2/\pi }{T}I.$$

*Thus, for all u with ∥u∥=1,*

$$V\left[{\hat{G}}^{*}\left(x\right)\cdot u\right]\leq V\left[\hat{G}\left(x\right)\cdot u\right]+\frac{1-2/\pi }{T}.$$

**Proof.**  It is straightforward to show that

$$\begin{array}{cc} \hat{G}\left(x\right) & :=\frac{1}{T}\sum_{t=1}^{T}\mathrm{sign}\left(w^{t}\cdot \eta^{t}\right)\left(\mu -w^{t}\right)\\ {\hat{G}}^{*}\left(x\right) & :=\frac{1}{T}\sum_{t=1}^{T}\frac{\eta^{t}}{∥\eta^{t}∥}\sqrt{\frac{2}{\pi }}\exp\left[-\frac{1}{2}{\left(\frac{\mu \cdot \eta^{t}}{∥\eta^{t}∥}\right)}^{2}\right]\\ \mathrm{Cov}[\hat{G}\left(x\right)] & =\frac{1}{T}\left(I-GG^{T}\right)\\ \mathrm{Cov}[{\hat{G}}^{*}\left(x\right)] & =\frac{1}{T}\left(E\left[\frac{\eta \eta^{T}}{{∥\eta ∥}^{2}}\frac{2}{\pi }e^{-{\left(\frac{\mu \cdot \eta }{∥\eta ∥}\right)}^{2}}\right]-GG^{T}\right) \end{array}$$

so for u≠0,

$$\begin{array}{cc} \; & Tu^{T}\left(\mathrm{Cov}\left[\hat{G}\left(x\right)\right]-\mathrm{Cov}\left[{\hat{G}}^{*}\left(x\right)\right]\right)u\\ \; & ={∥u∥}^{2}-\frac{2}{\pi }E\left[\frac{{\left|u\cdot \eta \right|}^{2}}{{∥\eta ∥}^{2}}e^{-{\left(\frac{\mu \cdot \eta }{∥\eta ∥}\right)}^{2}}\right]\geq {∥u∥}^{2}\left(1-\frac{2}{\pi }\right)>0. \end{array}$$

Above we have brought u inside the term with an expectation, which is then bounded using Cauchy–Schwarz on |u·η|/∥η∥≤∥u∥, and $e^{-|t|}\leq 1$ for all t∈R. □

### 4.2. All Sign Activations

Here we examine an important special case previously examined by Letarte et al. [6]: a feed-forward network with all sign activations and normal weights. This takes the form

$$f_{\theta }\left(x\right)=\mathrm{sign}\left(w_{L}\cdot \mathrm{sign}\left(W_{L-1}\cdots \mathrm{sign}\left(W_{1}x\right)\cdots \right)\right)$$

with $\theta :=\mathrm{vec}(w_{L},\cdots ,W_{1})$ and $W_{l}:={\left[w_{l,1}\cdots w_{l,d_{l}}\right]}^{T}$; l∈{1,...,L} are the layer indices. We choose unit-variance normal distributions on the weights, which factorise into $Q_{l}\left(W_{l}\right)=\prod_{i=1}^{d_{l}}q_{l,i}\left(w_{l,i}\right)$ with $q_{l,i}=N\left(\mu_{l,i},\;I_{d_{l-1}}\right)$. In the notation of Section 3, $\eta_{\theta^{\neg w}}\left(x\right)=\mathrm{sign}\left(W_{L-1}\cdots \mathrm{sign}\left(W_{1}x\right)\cdots \right)$ is the final layer activation, which could easily be sampled by mapping x through the first L−1 layers with draws from the weight distribution.

Instead, we go on to make an iterative replacement of the weight distributions on each layer by conditionals on the layer activations to obtain the summation

(9) $$\begin{array}{cc} \; & F_{Q}\left(x\right)=\sum_{a_{1}\in {\left\{+1,-1\right\}}^{d_{1}}}{\tilde{Q}}_{1}\left(a_{1}|x\right)\;\times \;\cdots \\ \cdots \; & \times \sum_{a_{L-1}\in {\left\{+1,-1\right\}}^{d_{L-1}}}{\tilde{Q}}_{L-1}\left(a_{L-1}|a_{L-2}\right)\;\mathrm{erf}\left(\frac{\mu_{L}\cdot a_{L-1}}{\sqrt{2}∥a_{L-1}∥}\right). \end{array}$$

The number of terms is exponential in the depth so we instead hierarchically sample the $a_{l}$. Like local reparameterisation, this leads to a considerable computational saving over sampling a separate weight matrix for every input. The conditionals can be found in closed form: we can factorise individual hidden units ${\tilde{Q}}_{l}\left(a_{l}|a_{l-1}\right):=\prod_{i=1}^{d_{l}}{\tilde{q}}_{l,i}\left(a_{l,i}|a_{l-1}\right)$, and find their activation distributions (with $a_{0}:=x$ and *z* a dummy variable):

$$\begin{array}{cc} {\tilde{q}}_{l,i}\left(a_{l,i}=\pm 1\;|a_{l-1}\right) & =\int_{0}^{\infty }N(z;\pm \mu_{l,i}\cdot a_{l-1},∥a_{l-1}{∥}^{2})\;dz\\ \; & =\frac{1}{2}\left[1\pm \mathrm{erf}\left(\frac{\mu_{l,i}\cdot a_{l-1}}{\sqrt{2}∥a_{l-1}∥}\right)\right]. \end{array}$$

A marginalised REINFORCE-style gradient estimator for *conditional* distributions can then be used; this does not necessarily have better statistical properties but in combination with the above is much more computationally efficient. This idea of “conditional sampling” is inspired by the local reinforce trick. Using samples ${\left\{\left(a_{1}^{t}\cdots a_{L-1}^{t}\right)\right\}}_{t=1}^{T}∼\tilde{Q}$,

(10) $$\frac{\partial F_{Q}\left(x\right)}{\partial \mu_{l,i}}\approx \frac{1}{T}\sum_{t=1}^{T}\mathrm{erf}\left(\frac{\mu_{L}\cdot a_{L-1}^{t}}{\sqrt{2}∥a_{L-1}^{t}∥}\right)\;\frac{\partial }{\partial \mu_{l,i}}\log{\tilde{q}}_{l,i}\left(a_{l,i}^{t}|a_{l-1}^{t}\right).$$

This formulation along with Equation (9) resembles the PBGNet model of [6], but derived in a very different way. Indeed both are equivalent in the single-hidden-layer case, but with more layers PBGNet uses an unusual tree-structured network to make the individual activations independent and avoid an exponential computational dependency on the depth in Equation (9). This makes the above summation exactly calculable but is also still not efficient enough in practice, so they resort further to a Monte Carlo approximation: informally, this draws new samples for every layer *l* based on an average of those from the previous layer, $a_{l}|{\left\{a_{l-1}^{\left(t\right)}\right\}}_{t=1}^{T}∼\frac{1}{T}\sum_{t=1}^{T}\tilde{Q}\left(a_{l}|a_{l-1}^{\left(t\right)}\right)$.

This is all justified within the tree-structured framework but leads to an exponential KL penalty which—as hinted by Letarte et al. [6] and shown empirically in Section 6—makes PAC–Bayes bound optimisation strongly favour shallower such networks. Our formulation avoids this, is more general—applying to alternative network structures—and we believe it is significantly easier to understand and use in practice.

## 5. PAC–Bayesian Objectives with Signed-Outputs

We now move to obtain binary classifiers with guarantees for the expected misclassification error, $R^{0-1}$, which we do by optimizing PAC–Bayesian bounds. Such bounds (as in Theorem 1) will usually involve the non-differentiable and non-convex misclassification loss $\ell_{0-1}$. However, to train a neural network we need to replace this by a differentiable surrogate, as discussed in the introduction.

Here we adopt a different approach by using our signed-output networks, where since f(x)∈{+1,−1}, there is an exact equivalence between the linear and misclassification losses, $\ell_{0-1}\left(f\left(x\right),y\right)=\ell_{\mathrm{lin}}\left(f\left(x\right),y\right)$, avoiding an extra factor of two from the inequality $\ell_{0-1}\leq 2\ell_{\mathrm{lin}}$.

Although we have only moved the non-differentiability into *f*, the form of a PAC–Bayesian bound and the linearity of the loss and expectation allow us to go further and aggregate,

(11) $$E_{f∼Q}\ell_{0-1}\left(f\left(x\right),y\right)=E_{f∼Q}\ell_{\mathrm{lin}}\left(f\left(x\right),y\right)=\ell_{\mathrm{lin}}\left(F_{Q}\left(x\right),y\right)$$

which allows us to use the tools discussed in Section 4 to obtain lower-variance estimates and gradients. Below we prove a small result to show the utility of this:

**Proposition** **5.** *Under the conditions of Proposition 3 and y∈{+1,−1},*

$$\begin{array}{cc} V_{Q}[ & \ell_{\mathrm{lin}}\left({\hat{F}}_{Q}^{*}\left(x\right),y\right)]\leq V_{Q}\left[\ell_{\mathrm{lin}}\left({\hat{F}}_{Q}\left(x\right),y\right)\right]\\ \; & \leq V_{f∼Q}\left[\ell_{0-1}\left(f\left(x\right),y\right)\right]=\frac{1}{4}\left(1-\right|F_{Q}\left(x\right){|}^{2}). \end{array}$$

**Proof.**  $$V_{Q}\left[\ell_{\mathrm{lin}}\left({\hat{F}}_{Q}\left(x\right),y\right)\right]=E_{Q}{\left|\frac{1}{2}\left(yF_{Q}\left(x\right)-y{\hat{F}}_{Q}\left(x\right)\right)\right|}^{2}=\frac{1}{4}V_{Q}\left[{\hat{F}}_{Q}\left(x\right)\right]$$

and a similar result for ${\hat{F}}_{Q}^{*}$. $f={\hat{F}}_{Q}$ if T=1 and $\ell_{\mathrm{lin}}\left(f\left(x\right),y\right)=\ell_{0-1}\left(f\left(x\right),y\right)$. The result then follows from this and Proposition 3. □

Combining (11) with Theorem 1, we obtain a directly optimizable, differentiable bound on the misclassification loss without introducing the above-mentioned factor of 2.

**Theorem** **2.** *Given P on θ and α>1, for all Q on θ and λ>1 simultaneously with probability at least 1−δ over $S∼D^{m}$,*

$$E_{\theta ∼Q}R^{0-1}\left(f_{\theta }\right)\leq \Phi_{\lambda /m}^{-1}\left[R_{S}^{\mathrm{lin}}\left(F_{Q}\right)+\frac{\alpha }{\lambda }\Delta \right]$$

*with $\Phi_{\gamma }^{-1}\left(t\right)=\frac{1-\exp(-\gamma t)}{1-\exp(-\gamma )}$, $f_{\theta }:R^{d}\to \left\{+1,-1\right\},\;\theta \in \Theta$, and $\Delta =\mathrm{KL}\left(Q|P\right)-\log\delta +2\log\left(\frac{\log\alpha^{2}\lambda }{\log\alpha }\right)$.*

Thus, for each λ, which can be held fixed (“**fix-λ**”) or simultaneously optimized throughout training for automatic regularisation tuning (“**optim-λ**”), we obtain a gradient descent objective:

(12) $$R_{S}^{\mathrm{lin}}\left({\hat{F}}_{Q}^{*}\right)+\frac{\mathrm{KL}(Q|P)}{\lambda }.$$

## 6. Experiments

All experiments (Table 1) run on “binary”-MNIST, dividing MNIST into two classes, of digits 0–4 and 5–9. Neural networks had three hidden layers with 100 units per layer and **sign**, sigmoid (**sgmd**) or **relu** activations, before a single-unit final layer with sign activation. *Q* was chosen as an isotropic, unit-variance normal distribution with initial means drawn from a truncated normal distribution of variance 0.05. The data-free prior *P* was fixed equal to the initial *Q*, as motivated by Dziugaite and Roy [4] (Section 5 and Appendix B).

The objectives **fix-λ** and **optim-λ** from Section 5 were used for batch-size 256 gradient descent with Adam [17] for 200 epochs. Every five epochs, the bound (for a minimising λ) was evaluated using the entire training set; the learning rate was then halved if the bound was unimproved from the previous two evaluations. The best hyperparameters were selected using the best bound achieved in these evaluations through a grid search of initial learning rates ∈{0.1,0.01,0.001}, sample sizes T∈{1,10,50,100}. Once these were selected training was repeated 10 times to obtain the values in Table 1.

λ in **optim-λ** was optimised through Theorem 2 on alternate mini-batches with SGD and a fixed learning rate of $10^{-4}$ (whilst still using the objective (12) to avoid effectively scaling the learning rate with respect to empirical loss by the varying λ). After preliminary experiments in **fix-λ**, we set λ=m=60,000, the training set size, as is common in Bayesian deep learning.

We also report the values of three baselines: **reinforce**, which uses the fix-λ objective without partial-aggregation, forcing the use of REINFORCE gradients everywhere; **mlp**, an unregularised non-stochastic relu neural network with tanh output activation; and the PBGNet model (**pbg**) from Letarte et al. [6]. For the latter, a misclassification error bound obtained through $\ell_{0-1}\leq 2\ell_{\mathrm{lin}}$ must be used as their test predictions were made through the sign of a prediction function ∈[−1,+1], not ∈{+1,−1}. Further, despite significant additional hyperparameter exploration, we were unable to train a three layer network through the PBGNet algorithm directly comparable to our method, likely because of the exponential KL penalty (in their Equation 17) within that framework; to enable comparison, we therefore allowed the number of hidden layers in this scenario to vary ∈{1,2,3}. Other baseline tuning and setup was similar to the above, see the Appendix A for more details.

During evaluation **reinforce** draws a new set of weights for every test example, equivalent to the evaluation of the other models; but doing so during training, with multiple parallel samples, is prohibitively expensive. Two different approaches to straightforward, not partially-aggregated, gradient estimation for this case suggest themselves, arising from different approximations to the *Q*-expected loss of the minibatch, B⊆S (with data indices B). From the identities

$$\begin{array}{cc} \nabla_{\phi }E_{\theta ∼q_{\phi }}R_{B}\left(f_{\theta }\right)\; & =E_{\theta ∼q_{\phi }}\frac{1}{\left|B\right|}\sum_{i\in B}\ell \left(f_{\theta }\left(x_{i}\right),y_{i}\right)\nabla_{\phi }\log q_{\phi }\left(\theta \right)\\ \; & =\frac{1}{\left|B\right|}\sum_{i\in B}E_{\theta ∼q_{\phi }}\ell \left(f_{\theta }\left(x_{i}\right),y_{i}\right)\nabla_{\phi }\log q_{\phi }\left(\theta \right) \end{array}$$

we obtain two slightly different estimators for $\nabla_{\phi }E_{\theta ∼q_{\phi }}R_{B}\left(f_{\theta }\right)$:

$$\begin{array}{cc} \; & \frac{1}{T|B|}\sum_{t=1}^{T}\sum_{i\in B}\ell \left(f_{\theta^{\left(t,i\right)}}\left(x_{i}\right),y_{i}\right)\nabla_{\phi }\log q_{\phi }\left(\theta^{\left(t,i\right)}\right)\\ \; & \frac{1}{T|B|}\sum_{i\in B}\sum_{t=1}^{T}\ell \left(f_{\theta^{t}}\left(x_{i}\right),y_{i}\right)\nabla_{\phi }\log q_{\phi }\left(\theta^{t}\right). \end{array}$$

The first draws many more samples and has lower variance but is much slower computationally; even aside from the O(|B|) increase in computation, there is a slowdown as the optimised BLAS matrix routines cannot be used, and the very large matrices involved may not fit in memory (see for more information [16]).

Therefore, as is standard in the Bayesian Neural Network literature with the pathwise estimator, we use the latter formulation, which has a similar computational complexity to local-reparameterisation and our marginalised REINFORCE estimator (10). We should note though that in preliminary experiments, the alternate estimator did not appear to lead to improved results. This clarifies the advantages of marginalised sampling, which can lead to lower variance with a similar computational cost.

## 7. Discussion

The experiments demonstrate that partial-aggregation enables training of multi-layer non-differentiable neural networks in a PAC–Bayesian context, which is not possible with REINFORCE gradients and a multiple-hidden-layer PBGNet [6]. These obtained only vacuous bounds, and our misclassification bounds also improve those of a single-hidden-layer PBGNet.

Our experiments raise a couple of questions: firstly, why is it that lower variance estimates empirically lead to tighter bounds? We speculate that the faster convergence of SGD in this case takes us to a more “local” minimum of the objective, closer to our initialisation. Since most existing PAC–Bayes bounds for neural networks have a very strong dependence on this distance from initialisation through the KL term, this leads to tighter bounds. This distance could also be reduced through other methods we consider out-of-scope, such as the data-dependent bounds employed by Dziugaite and Roy [18] and Letarte et al. [6].

A second and harder question is asking why the non-stochastic mlp model obtains a lower overall error. The bound optimisation is empirically quite conservative, but does not necessarily lead to better generalisation; understanding this gap is a key question in the theory of deep learning.

In future work we will develop significant new tools to extend partial-aggregation to multi-class classification, and to improve test prediction bounds for $\mathrm{sign}({\hat{F}}_{Q}\left(x\right))$ with T>1, as in PBGNet, which gave slightly improved predictive performance despite the inferior theoretical guarantees.

## Figures and Tables

**Table 1:** Average (from ten runs) binary-MNIST losses and bounds (δ=0.05) for the best epoch and optimal hyperparameter settings of various algorithms. Hyperparameters and epochs were chosen by bound if available and non-vacuous, otherwise by training linear loss. Bold numbers indicate the best values and standard deviation is reported in italics.

	mlp	pbg	Reinforce		Fix-λ			Optim-λ		
			sign	relu	sign	sgmd	relu	sign	sgmd	relu
**Train Linear**	**0.78**	8.72	26.0	18.6	8.77	7.60	6.35	6.71	6.47	5.41
*error, 1σ*	*0.08*	*0.08*	*0.8*	*1.4*	*0.04*	*0.19*	*0.10*	*0.11*	*0.18*	*0.16*
**Test 0–1**	**1.82**	5.26	25.4	17.9	8.73	7.88	6.51	6.85	6.84	5.61
*error, 1σ*	*0.16*	*0.18*	*1.0*	*1.5*	*0.23*	*0.30*	*0.19*	*0.27*	*0.21*	*0.20*
**Bound 0–1**	-	40.8	100	100	21.7	18.8	**15.5**	22.6	19.3	16.0
*error, 1σ*	*-*	*0.2*	*0.0*	*0.0*	*0.04*	*0.17*	*0.04*	*0.03*	*0.31*	*0.05*

## Data Availability

Not applicable

## Appendix A. Further Experimental Details

### Appendix A.1. Aggregating Biases with the Sign Function

We used a bias term in our network layers, leading to a simple extension of the above formulation, omitted in the main text for conciseness:

$$E_{w∼N\left(\mu ,\Sigma \right),b∼N\left(\beta ,\sigma^{2}\right)}\;\mathrm{sign}\left(w\cdot x+b\right)=\mathrm{erf}\left(\frac{\mu \cdot x+\beta }{\sqrt{2(x^{T}\Sigma x+\sigma^{2})}}\right)$$

since $w\cdot x+b∼N(\mu \cdot x+\beta ,x^{T}\Sigma x+\sigma^{2})$ and

$$\begin{array}{cc} E_{z∼N(\alpha ,\beta^{2})}\mathrm{sign}z & =P(z\geq 0)-P(z<0)\\ \; & =[1-\Phi (-\alpha /\beta )]-\Phi (-\alpha /\beta )\\ \; & =2\Phi \left(\alpha /\beta \right)-1=\mathrm{erf}\left(\alpha /\sqrt{2}\beta \right). \end{array}$$

The bias and weight co-variances were chosen to be diagonal with a scale of 1, which leads to some simplification in the above.

### Appendix A.2. Dataset Details

We used the MNIST dataset version 3.0.1, available online at http://yann.lecun.com/exdb/mnist/ (accessed on 4 June 2021), which contains 60,000 training examples and 10,000 test examples, which were used without any further split, and rescaled to lie in the range [0,1]. For the “binary”-MINST task, the labels +1 and −1 were assigned to digits in {5,6,7,8,9} and {0,1,2,3,4}, respectively, and images were scaled into the interval [0,1].

### Appendix A.3. Hyperparameter Search for Baselines

The baseline comparison values offered with our experiments were optimized similarly to the above, for completeness we report everything here.

The MLP model had three hidden ReLu layers of size 100 each trained with Adam, a learning rate ∈{0.1,0.01,0.001} and a batch size of 256 for 100 epochs. Complete test and train evaluation was performed after every epoch, and in the absence of a bound, the model and epoch with lowest train linear loss was selected.

For PBGNet we choose the values of hyperparameters from within these values giving the least bound value. Note that, unlike in [6], we do not allow the hidden size to vary {∈10,50,100}, and we use the entire MNIST training set as we do not need a validation set. While attempting to train a three hidden layer network, we also searched through the hyperparameter settings with a batch size of 64 as in the original, but after this failed, we returned to the original batch size of 256 with Adam. All experiments were performed using the code from the original paper, available at https://github.com/gletarte/dichotomize-and-generalize (accessed on 4 June 2021).

Since we were unable to train a multiple-hidden-layer network through the PBGNet algorithm, for this model only we explored different numbers of hidden layers ∈{1,2,3}.

### Appendix A.4. Final Hyperparameter Settings

In Table A1 we report the hyperparameter settings used for the experiments in Table 1 after exploration. To save computation, hyperparameter settings that were not learning (defined as having a whole-train-set linear loss of >0.45 after ten epochs) were terminated early. This was also done on the later evaluation runs, where in a few instances the fix-λ sigmoid network failed to train after ten epochs; to handle this we reset the network to obtain the main experimental results.

**Table A1:** Chosen hyperparameter settings and additional details for results in Table 1. Best hyperparameters were chosen by bound if available and non-vacuous, otherwise by best training linear loss through a grid search as described in Section 6 and Appendix A.3. Run times are rounded to nearest 5 min.

	mlp	pbg	Reinforce		Fix-λ			Optim-λ		
			sign	relu	sign	relu	sgmd	sign	relu	sgmd
Init. LR	0.001	0.01	0.1	0.1	0.01	0.1	0.1	0.01	0.1	0.1
Samples, T	-	100	100	100	100	50	10	100	100	10
Hid. Layers	3	1	3	3	3	3	3	3	3	3
Hid. Size	100	100	100	100	100	100	100	100	100	100
Mean KL	-	2658	15,020	13,613	2363	3571	3011	5561	3204	4000
Runtime/min	10	5	40	40	35	30	25	35	30	25

For clarity we repeat here the hyperparameter settings and search space:

- Initial Learning Rate ∈{0.1,0.01,0.001}.
- Training Samples ∈{1,10,50,100}.
- Hidden Size =100.
- Batch Size =256.
- Fix-λ, λ=m=60,000.
- Number of Hidden Layers =3 for all models, except PBGNet ∈{1,2,3}.

### Appendix A.5. Implementation and Runtime

Experiments were implemented using Python and the TensorFlow library [19]. Reported approximate runtimes are for execution on a NVIDIA GeForce RTX 2080 Ti GPU.
